# Correlation Analysis of Linear Viscoelastic (LVE) Properties, Damage Resistance and Microstructure of Unmodified Asphalt Binders with the Same Penetration Grade

**DOI:** 10.3390/ma15217709

**Published:** 2022-11-02

**Authors:** Chao Wang, Lihao Song, Zhen Wang, Yifang Chen, Bochao Zhou

**Affiliations:** 1 Department of Road & Railway Engineering, Beijing University of Technology, Beijing 100124, China; 2 Beijing Municipal Road and Bridge Building Materials Group Co., Ltd., Beijing 100176, China

**Keywords:** asphalt binder, rheological performance, linear viscoelasticity, microstructure, correlation analysis

## Abstract

The penetration grade system is still widely adopted for selecting asphalt binder with desired paving performance. However, the initial material compositions of asphalt binder with the same penetration level are still different, and vary with the crude oil source and essentially result in different rheological performance. This study aimed to assess the linear viscoelastic (LVE) properties, and high- and intermediate-temperature and microscale characteristics of seven unmodified asphalt binders from different sources and countries with the same penetration level of 70. The LVE parameters were firstly evaluated followed by comparisons to various damage-based indexes. The microstructure of asphalt binders was further investigated followed by correlations between morphology and performance parameters. Experimental results indicate the |G*|/sin *δ* is well related to the MSCR-based non-recoverable creep compliance; furthermore, the *R* and |G*|·sin *δ* can generally represent the LAS-based failure strain and fatigue life, respectively. The viscoelastic nature of tested binders was clearly distinguished and related to rheological performance by atomic force microscopy (AFM). The roughness parameters and the phases’ content derived from AFM images showed significant correlations with LVE characteristics and fatigue resistance nature, respectively. This research provides theoretical foundations for further investigating the rheological performance and microstructure characteristics, and their correlations with asphalt binders.

## 1. Introduction

Fatigue cracking, rutting, and thermal cracking are the common types of distress of asphalt pavements in the field [1,2,3]. It is known that the material properties of hot mix asphalt (HMA) have a significant role in achieving better pavement performance. Moreover, the asphalt binder, which provides the only viscoelastic origin of HMA, has various impacts on the performance of HMA and pavements [4]. Thus, the performance characterization and prediction of asphalt binder has been widely addressed in pavement material research. The penetration grade, which represents the binder stiffness, consistency, shear resistance, and the relative viscosity under a given temperature, is still widely utilized as the specification parameter to grade the asphalt binder in China and many other countries. However, the penetration grade-based performance ranking of asphalt binders is poorly correlated to the HMA and field pavement performance [5]. Therefore, numerous performance-based/related testing procedures, specifications, and parameters for asphalt binders have been thoroughly investigated in the past 30 years.

During the Strategic Highway Research Program (SHRP) project in the United States in the early 1990s, the specification based on the performance grade (PG) was then established, verified, and applied for asphalt binder [6]. Furthermore, the dynamic shear rheometer (DSR) was first utilized to measure the rheological performance of asphalt binder. Linear viscoelastic (LVE) characteristics and various related parameters of asphalt binder can be easily obtained through a single point dynamic testing under a small loading level at a desired temperature, of which the material parameters of the dynamic shear modulus (|G*|) and phase angle (*δ*) are the two most fundamental. According to the SHRP PG specification, the parameters of |G*|/sin *δ* and |G*|·sin *δ* are calculated from |G*| and *δ*, and utilized as the evaluation indexes of rutting potential and fatigue resistance of asphalt binder. The physical meaning of |G*|·sin *δ* is the viscous component in |G*| (i.e., the dynamic shear modulus) and it directly represents the dissipated strain energy during the dynamic loading cycles [7,8]. However, the subsequent verification works demonstrated that the performance of asphalt mixture and pavement in the field generally cannot be revealed very well from the binder parameters (|G*|/sin *δ*, |G*|·sin *δ*, etc.). Thus, in recent years, a large amount of research has been conducted to develop the new damage-based standard tests of asphalt binders [9]. The Multiple Stress Creep Recovery (MSCR) test (AASHTO T 350-14) was developed by the U. S. federal highway administration (FHWA) as a specification evaluation procedure for the high-temperature performance of asphalt binder. Significant correlations were observed between MSCR based binder indexes and the high-temperature performance of asphalt mixtures and field pavements [10,11,12]. Regarding asphalt binder fatigue, the linear amplitude sweep (LAS) test, an accelerated fatigue procedure, was developed by Johnson et al. for evaluating the fatigue resistance of asphalt binder under cyclic loading (AASHTO TP 101) [13,14,15,16]. In cooperation with the viscoelastic continuum damage (VECD) model, the fatigue life of asphalt binder under constant strain amplitude fatigue loading can be simulated and predicted [17,18,19,20]. Recent research has been conducted and calibrated to increase the accuracy of the LAS-based fatigue life prediction by establishing a unified failure criterion [21,22,23]. Since the LVE parameters and damage-based indexes are popularly utilized, it is necessary to establish the link between them and illustrate the suitable situations for applying them.

Moreover, it is worth mentioning that even asphalt binder with the same penetration grade has distinguished rheological properties. The difference essentially originates from the composition of the material and the resultant microscopic structure. To study the micro-nature of asphalt binder, various technologies have been applied to investigate its material composition and resultant microstructure characteristics. In contrast to other technologies, atomic force microscopy (AFM) is used worldwide due to the high-resolution identification of material composition and the quantitative analysis of the microstructure characteristics [24]. In addition, the sample preparation of AFM is also simple. In 1996, Lober et al. first introduced AFM to asphalt binder and observed the well-known “bee structure”, and regarded it as a composition of asphaltenes [25]. In the past 20 years, the chemical nature and formation mechanism of the bee structure was comprehensively studied. Masson et al. suggested the bee structure is formed by the combination between the cationic of nickel and vanadium compounds (commonly present in asphaltenes) and the aromatic compounds [26]. Hung et al. proved the effect of paraffin wax thin film on the bee structure, and proposed the process of the formation [27]. Magonov et al. considered that the bee structure is mainly composed of wax and a variety of alkanes under the effect of surface wrinkling [28]. Some research showed that the coprecipitation of wax and asphaltene, in addition to other fractions of asphalt, results in the formation of the bee structure [29,30]. Furthermore, external factors such as asphalt type, aging, modification, and thermal history largely impact the microstructure (i.e., the size and number of bee structures), as well as the rheological performance. Dokandari et al. found that the reclaimed asphalt resulted in unreliable rheological performance with no bee structure observed; however, the rheological performance improved and the bee structure reappeared after the addition of waste oil as rejuvenators [31]. Zhang et al. focused on the effect of aging on the bee structure. The results illustrated that the increasing aging process decreased the number of bee structures, but the distribution was more dispersed [32]. Li et al. found that the modification effect of graphene facilitated the nucleation of bee structures, which led to bee structures with a larger number but smaller volume. Moreover, the anti-aging ability improved after the incorporation of graphene [33]. Nahar et al. found that the microstructure characteristics were dependent on the maximum hold temperature of the bitumen and a “reset temperature” temperature hypothesis was proposed to explain the different microstructures obtained from various thermal annealing or quenching procedures [34]. Ābele et al. focused on the effect of modification and RTFOT aging on the rheological properties. The result showed that the rapeseed oil biodiesel-modified RTFOT asphalt binder demonstrated improved rutting and fatigue resistance [35]. As a review of the literature suggests, limited works have paid attention to the quantitative analysis on the microstructure, and the effects of material composition and resultant microstructure on the rheological properties are also lacking.

In this study, the DSR and AFM tests were carried out and the specific objectives of this comparative study were to: (1) evaluate the rheological characteristics of several unmodified asphalt binders and identify the possible performance relationship between the LVE parameters and damage-based indexes; (2) investigate the microstructure of tested unmodified asphalt binders and establish the potential correlation between morphology parameters and performance parameters.

## 2. Materials and Methods

### 2.1. Materials

In this study, the materials for testing included seven unmodified asphalt binders from different crude oil sources, and countries with the same penetration grade of 70 were selected. These employed asphalt binders are separately denoted as A, B, C, D, E, F, and G in this work in order to avoid any commercial purpose, and the physical properties of all tested binders are shown in Table 1.

### 2.2. Rheology Investigation

The linear viscoelastic (LVE) behavior, high-temperature rutting potential, and intermediate-temperature fatigue performance of seven asphalt binders were evaluated by the FS, MSCR, and LAS tests, respectively, and the corresponding LVE parameters and damage-based indexes were obtained. An Anton Paar MCR 302 dynamic shear rheometer (DSR) was employed in this study with two differently sized parallel plates of 25 mm and 8 mm for high-temperature condition and intermediate-temperature condition, respectively. At least two sample replicates were completed for each test. If the variation between the two replicates was distinguished, other replicates were run to ensure the coefficients of variation were within 10%. The testing procedures are summarized as follows.

#### 2.2.1. Frequency Sweep Test

The undamaged LVE behavior and related parameters were obtained by the frequency sweep (FS) test. Mechanical responses of dynamic shear modulus (|G*|) at different loading frequency under a single given temperature can be obtained based on dynamic shear tests. For asphalt binder material, a higher |G*| indicates a higher level of stiffness. In this study, the FS test was conducted at a constant strain of 0.1% varying the frequencies from 0.1 to 100 rad/s. A wide range of temperature from 10 to 70 °C (with an increase of 10 °C) was set to measure the LVE behavior and various LVE parameters. By using the time–temperature superposition principle (TTSP), the obtained |G*| values at various conditions were further horizontally shifted together to the reference temperature (set as 20 °C in this study) and the mastercurves of the dynamic shear modulus were then constructed by applying the Christenson–Anderson (CA) model as shown in Equation (1):

(1) $$\left|G^{*}(\omega_{r})\right|=G_{g}{\left[1+{\left(\frac{\omega_{c}}{\omega_{r}}\right)}^{\left(\log2\right)/R}\right]}^{-R/\log2}$$

where *G*_g_ is the glassy modulus taken as 1 GPa, *ω*_c_ is the crossover angular frequency, *ω*_r_ is the reduced angular frequency and calculated as *ω*_r_ = *ω* × *ϕ*_T_, *ω* is the physical angular frequency, *ϕ*_T_ is the time–temperature shift factor fitted via Equation (2):

(2) $$\log\left(\phi_{T}\right)=a_{1}{\left(T-T_{R}\right)}^{2}+a_{2}\left(T-T_{R}\right)$$

where *a*_1_ and *a*_2_ are the fitting parameters of the TTSP shift factor function and *T*_R_ is the reference temperature.

#### 2.2.2. Multiple Stress Creep Recovery Test

The high-temperature rutting resistance was accessed by the multiple stress creep recovery (MSCR) test at 60 °C following the AASHTO Standard T 350-14 [10]. The MSCR test was carried out under 0.1 and 3.2 kPa, and the time–strain curves were directly obtained from test data to preliminarily analyze the shear strain level. The non-recoverable creep compliance (*J*_nr_) was further obtained to fully reveal the residual strain after the creep and recovery cycles, and a lower *J*_nr_ value means better high-temperature performance. Nowadays, the *J*_nr_ value at 3.2 kPa (*J*_nr3.2_) is widely recognized and utilized to characterize and distinguish the high-temperature rutting potential among various types of asphalt materials.

#### 2.2.3. Linear Amplitude Sweep Test

The intermediate-temperature fatigue resistance was measured by the linear amplitude sweep (LAS) test at 20 °C following the AASHTO TP 101-14 [13]. Firstly, a frequency sweep test was designed to stabilize the sample, and a strain amplitude sweep test was secondly carried out to measure the fatigue damage resistance of asphalt binders. The simplified-viscoelastic continuum damage (S-VECD) model was applied in this study to analysis the LAS test data [21,22,23]. The failure identification during the LAS test is determined by an energy-based approach and the fatigue life is also simulated based on the S-VECD analysis framework [21].

### 2.3. Microstructure Investigation

#### 2.3.1. Sample Preparation

**Figure 1:**
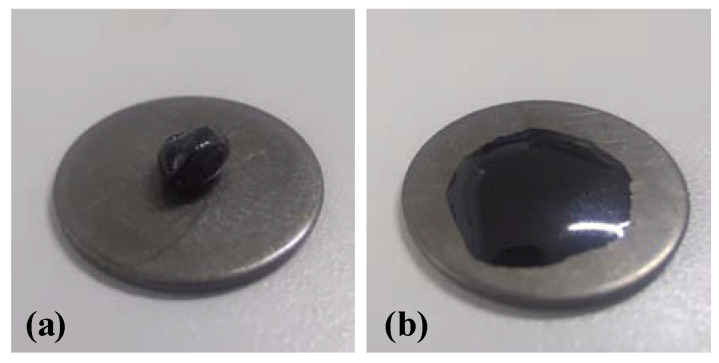
Samples (**a**) before and (**b**) after heating.

A small piece of asphalt binder was cut and placed at the center of a circular plate with a diameter of 15 mm. Then, the plate and asphalt were heated in an oven at 120 °C for 15 min to avoid overaging. During this step, the asphalt distributed evenly and formed a thin film. Finally, the sample was kept under the room temperature until totally cooled. The prepared samples are shown in Figure 1.

#### 2.3.2. Topography and Morphology Test

An Asylum Research MFP-3D atomic force microscope (AFM) was employed in this study to investigate the microscale characteristics for seven unmodified asphalt binders under room temperature. A silicon probe with 70 kHz. resonant frequency and 2 N/m elastic coefficient was selected. The topography and phase images were recorded in the tapping mode under the repulsive state with a range of 20 μm × 20 μm, and typical bee structures could be seen in most images. The morphology parameters of tested samples were also calculated from the AFM images through Nano analysis and Image pro-plus to assess further microscale characteristics and perform correlation analyses.

## 3. Results and Discussions

### 3.1. Linear Viscoelastic Characteristics

#### 3.1.1. Dynamic Shear Modulus Mastercurves

**Figure 2:**
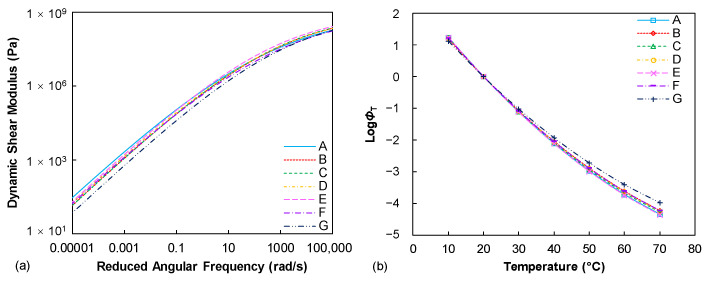
Frequency sweep test results (reference temperature: 20 °C): (**a**) dynamic shear modulus mastercurves; (**b**) TTSP shift factors.

Figure 2a summaries the dynamic shear modulus mastercurves of seven tested binders. Generally, it is observed that these binders show a similar level of dynamic modulus values across the various loading frequencies. This is expected since the penetration grades of those binders are identical. The penetration depth basically reflects the stiffness level of the asphalt binder at a specific temperature condition and the dynamic modulus is also a viscoelastic indicator of the binder stiffness at various loading frequencies and temperatures. However, slight differences can still be found from the dynamic modulus mastercurves, especially at the lower frequency (higher temperature) conditions. The binder A is identified as a stiffer binder when decreasing the loading frequencies while the binder G relatively shows the lowest modulus level. The temperature sensitivity of the tested binders can be described from the temperature shift factor (*ϕ*_T_) results, as compared in Figure 2b. It can be observed that the *ϕ*_T_ results of the seven binders at lower temperature range are identical to each other, whereas they gradually exhibit discrepancies with the enhanced temperature conditions, especially for the binder G.

#### 3.1.2. Linear Viscoelastic Properties

Various parameters including *R*, *ω*_c_, G-R, |G*|/sin *δ*, and |G*|·sin *δ* parameters were further utilized to assess the LVE performance of the tested binders. The rheological indexes *R* and *ω*_c_ that were obtained from the CA model-based dynamic modulus mastercurve are very meaningful parameters for the viscoelastic material characterization. The shape factor of *R* is proportional to the width of the binder relaxation spectrum and also bound to the degree of skewness in the spectrum. A higher value of *R* means that the asphalt becomes less viscous and more brittle at moderate loading times and intermediate temperatures [36]. Rowe previously also demonstrated a strong relationship between the *R* values and the material fatigue life [37]. The *ω*_c_ (i.e., the crossover frequency) is graphically the frequency where the storage modulus and loss modulus cross, and it measures the overall binder hardness and stiffness. Additionally, the parameter of Glover–Rowe (G-R) was recently introduced as a potential index of asphalt binder cracking resistance and verified to be well correlated with the binder ductility property [38]. The lower the G-R value, the better the crack resistant of asphalt binder, and the value of G-R was calculated according to the Equation (3) at 15 °C and 0.005 rad/s from the fitted dynamic shear modulus mastercurves in this study. Moreover, |G*|/sin *δ* and |G*|·sin *δ* at 10 rad/s under a small strain were calculated to access the rutting potential and fatigue resistance of asphalt binder within the LVE domain in the SHRP binder specification. In this study, the |G*|/sin *δ* and |G*|·sin *δ* under 10 rad/s were respectively derived at 60 and 20 °C from the FS tests.

(3) $$G\text{-}R=\frac{\left|G^{*}\right|\cos^{2}\delta }{\sin\delta }$$

**Figure 3:**
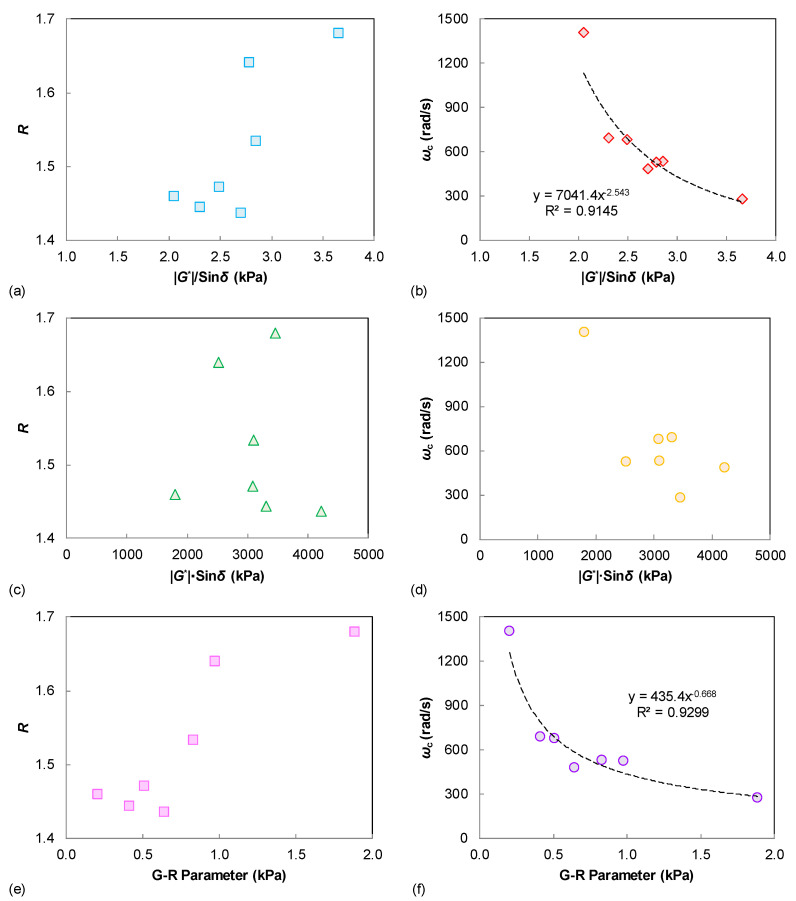
Relationships between the linear viscoelastic parameters: (**a**) |G*|/sin *δ* vs. *R*; (**b**) |G*|/sin *δ* vs. *ω*_c_; (**c**) |G*|·sin *δ* vs. *R*; (**d**) |G*|·sin *δ* vs. *ω*_c_; (**e**) G-R parameter vs. *R*; (**f**) G-R parameter vs. *ω_c_*.

Figure 3 summarizes the possible relationship between these LVE parameters of the tested binders. The rheological indexes *R* and *ω*_c_ are respectively compared to the binder rutting and fatigue indexes. It is observed that the shape factor of *R* only shows a similar trend to the binder |G*|/sin *δ* and G-R parameters. However, the crossover frequency of *ω*_c_ is well correlated to the |G*|/sin *δ* and G-R parameters, and also shows an identical trend with the |G*|·sin *δ* results. This can be explained because the *ω*_c_ is a parameter that is related to the binder stiffness, whereas the LVE rutting and fatigue parameters (G*|/sin *δ*, |G*|·sin *δ*, and G-R parameters) are also significantly affected by the stiffness level. A validation of those LVE performance correlations is further investigated using the damage-based indexes as described in later sections.

### 3.2. Damage Resistance Characteristics

#### 3.2.1. MSCR Test Results

**Figure 4:**
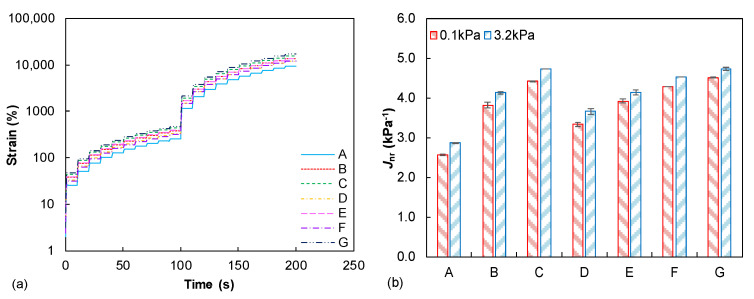
MSCR test results: (**a**) recorded time–strain curves; (**b**) comparison of *J_nr_* values.

Figure 4a summarizes the recorded time–strain responses of the tested binders from the MSCR test. It is clearly observed that the binder A shows the lowest non-recovered deformation, whereas the binder G displays the largest permanent deformation. Meanwhile, less distinguished time–strain curves are observed for the five other tested binders. Generally, the binder ranking regarding the rutting resistance is consistent with the dynamic shear modulus mastercurves as previously discussed in Figure 2a, indicating that the rutting potential of asphalt binder is positively related to its stiffness level. Nowadays, the *J*_nr_ parameter under 3.2 kPa is utilized as the specification index to access the asphalt binder rutting resistance. Figure 4b summarizes and compares the calculated *J*_nr_ values under two stress levels of tested asphalt binders. The *J*_nr_ values of binder A are the smallest at both stress levels, suggesting its best rutting resistance again among the seven binders. In addition, binders G and C exhibit higher *J*_nr3.2_ values than the other tested binders, which indicate their larger rutting potential under repeated traffic loading. Therefore, the *J*_nr_-based evaluation for permanent deformation resistance of tested binders generally keeps the identical trend to the previous dynamic modulus and time–strain response analyses. In summary, it can be briefly concluded that with the same penetration grade of seven unmodified asphalt binders, their rutting resistance is still obviously distinguished from each other and, thus, the damage-based protocol and related evaluation parameter are able to effectively identify their specific performance under high-temperature conditions.

**Figure 5:**
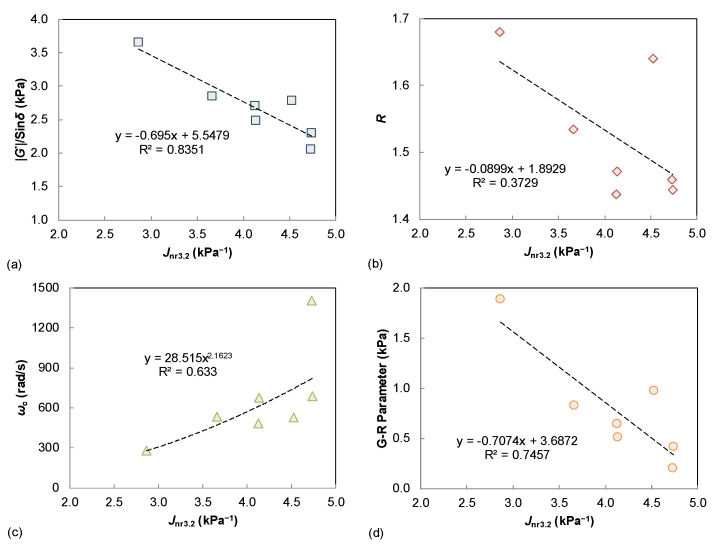
Comparison of *J*_nr3.2_ and LVE parameters: (**a**) |G*|/sin *δ*; (**b**) *R*; (**c**) *ω*_c_; (**d**) G-R parameter.

The MSCR-based *J*_nr3.2_ value is compared to the |G*|/sin *δ*, *R*, *ω*_c_, and G-R in Figure 5. The first notable observation is a good linear correlation between *J*_nr3.2_ and |G*|/sin *δ* (R^2^ > 0.8) as shown in Figure 5a. It is well known that the SHRP parameter of |G*|/sin *δ* was proposed as the binder rutting index based on the unmodified asphalt materials, and the MSCR procedure was developed mainly for the modified binders and purchase specification of blind modification. Therefore, |G*|/sin *δ* is still an effective performance parameter for the rutting potential of asphalt binder when only evaluating the neat binders. In addition, the two rheological parameters of *R* and *ω*_c_ are not well related to the binder *J*_nr3.2_ values, whereas the G-R parameter displays a promising trend. As previously discussed, the G-R parameter is also defined based on the dynamic shear modulus (|G*|) and phase angle (*δ*), indicating a reasonable link to the binder G*|/sin *δ* parameter.

#### 3.2.2. LAS Test Results

**Figure 6:**
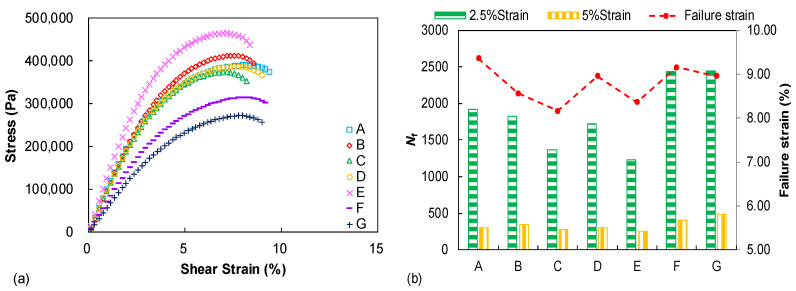
LAS test results: (**a**) stress–strain curves; (**b**) failure strain comparison and strain-controlled fatigue life prediction.

Figure 6a presents the measured stress–strain curves during the LAS test and each curve is displayed up to the identified failure occurrence. The binder E shows the highest peak stress amplitude whereas the binder G exhibits the lowest one. However, it should be kept in mind that the peak stress amplitude does not represent the fatigue potential but only indicates binder stress response under the strain–sweep loading. Figure 6b summarizes the identified failure strain (*γ*_f_) and predicted fatigue life (*N*_f_) values from the LAS test, in which distinguished failure properties can be observed among the tested binders. Binders A and C, respectively, display the highest and lowest failure strain values. It was previously demonstrated that the failure strain parameter only reveals the binder strain tolerance/flexibility with increasing the strain amplitude in the LAS test and, thus, it is related to the binder fatigue property to some degree but not the full fatigue performance [23]. The strain-controlled fatigue life, which is regarded as a direct and easily understood parameter to reflect fatigue resistance, is simulated under two strain levels (2.5% and 5%) for all tested binders. It can be observed that the tested binders exhibit obvious discrepancies on the fatigue performance and the *N*_f_ -based ranking is slightly different to the failure strain evaluation. Binders C and E still show relatively low *N*_f_ levels but the fatigue performance of binder F and G is obviously better than that of other binders, especially binder A, which previously showed the highest failure strain. Therefore, it is concluded that the fatigue resistance of the seven unmodified binders with the same penetration grade are still distinguished from each other and the damage-based procedure is needed to achieve a more accurate fatigue characterization.

**Figure 7:**
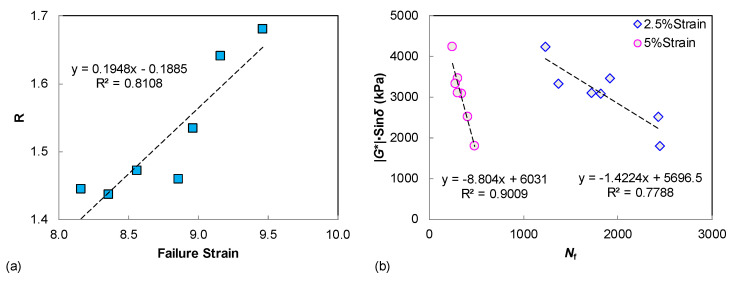
The relationships between LAS results and LVE parameters: (**a**) failure strain vs. *R*; (**b**) *N*_f_ vs. |G*|·sin *δ*.

The S-VECD fatigue modeling is established for the LAS test data interpretation, so it is necessary and helpful to investigate the possible relationship between LAS-based asphalt fatigue resistance and the LVE parameters, since only unmodified neat binders are covered in this study. As seen in Figure 7a, a fairly good correlation was verified for the rheological index of *R* and the LAS failure strain. This can be explained because the shape factor of *R* is related to the width of the binder relaxation spectrum. Furthermore, the LAS failure strain describes the binder strain tolerance and flexibility, which also reveal the relaxation characteristics to some degree. Additionally, the |G*|·sin *δ* parameter is found to be well correlated to the LAS-based binder fatigue life, under either 2.5% or 5% strain amplitudes, as shown in Figure 7b, suggesting that the SHRP parameter of |G*|·sin *δ* can still provide a reasonable assessment of binder fatigue resistance when only unmodified asphalts are compared. However, damage-based procedures such as the MSCR and LAS tests are still recommended for binder rutting and fatigue performance characterization, especially when covering the modified/hybrid modified asphalt.

### 3.3. Microstructure Characteristics

#### 3.3.1. Topography and Morphology Test Results

**Figure 8:**
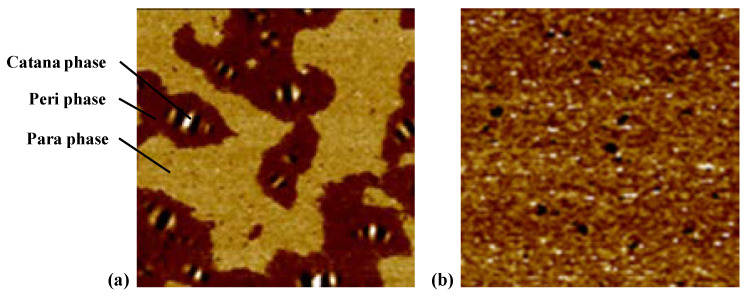
Schematic diagrams for different microstructures: (**a**) three phases; (**b**) a continuous phase.

Two different types of asphalt binders were classified based on the morphology of AFM phase images and the schematic diagrams are shown in Figure 8. As seen from Figure 8a, three phases are observed, i.e., the catana phase for the bee structures in the topography image, the peri phase for the dark area adjacent to the catana phase, and the para phase for the light area peripheral to the peri phase. Figure 8b shows an almost continuous phase with separated domains of different sizes instead of the bee structure.

**Figure 9:**
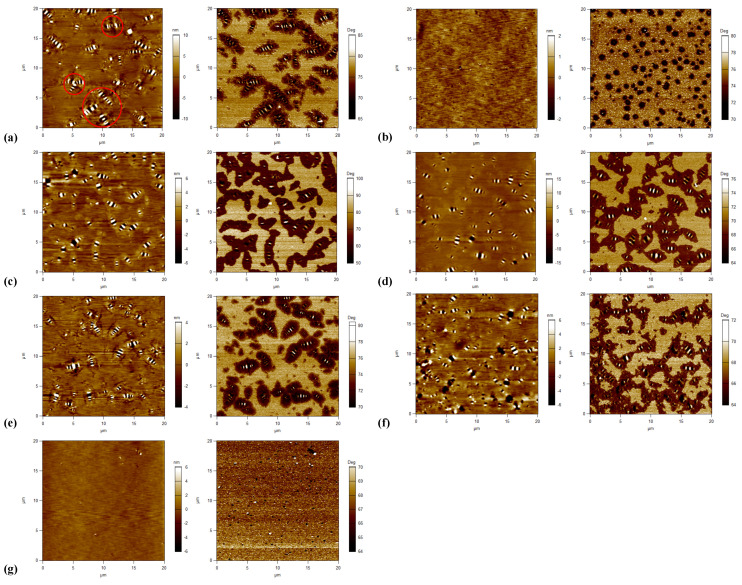
Topography (**left**) and phase (**right**) images of tested asphalt binders: (**a**) A; (**b**) B; (**c**) C; (**d**) D; (**e**) E; (**f**) F; (**g**) G.

The topography and phase images of tested asphalt binders are shown in Figure 9. Five of seven asphalt binders show three phases with a bee structure and another two asphalt binders show another microstructure without a bee structure. For asphalt binders with a bee structure (i.e., binders A, C, D, E, and F), binder A performs a little differently, since some bee structures are not independent of one another in the topography image. Parts of them are gathered together and form multiarm star-shapes (as shown in Figure 9a), which reveals a strong interaction between crystalline waxes and the remaining non-wax components. The special surface topography is probably associated with the viscoelastic nature of the asphalt binder, which could be explained by diffusion theory [39]. According to the diffusion theory, the higher the elasticity of asphalt binder, the weaker the resistant force on migration. Due to the weak resistant force, the migration of particles is unrestricted, thus leading to congregated and big bee structures. Thus, binder A is proved to be a relatively stronger, stiffer, and more elastic material, which is consistent with the results of the FS and MSCR tests (i.e., the highest dynamic shear modulus, the lowest non-recovered deformation and the lowest *J*_nr_ value). Other binders with a bee structure show a similar viscoelastic nature, since they all show a general microstructure with intact, separate, and identifiable bee structures, in addition to a flat area.

As seen in Figure 9, for binders with an almost continuous phase (i.e., binders B and G), the typical bee structures are lost and they are replaced by a smooth surface. This is largely related to the wax content of asphalt binder, since the long-chain alkyl of asphaltene fails to eutectic with wax, resulting in difficult formation of the crystal nucleus of the bee structure [29,30]. In the phase image of binder B, an almost continuous phase with light and dark quasi-spherical domains ranging from nano-level to micron-level are observed. Compared to binder B, binder G has more light domains which bring viscous behavior into the asphalt, and fewer dark domains, which decrease the rigidity and solidity of asphalt, respectively. Based on the analyses of the micro-morphology, binder G is considered to be a relatively viscous and soft material, and leads to specific rheological performance (i.e., the lowest dynamic shear modulus, |G*|·sin *δ*, G-R, and |G*|/sin *δ*, and the highest *ω*_c_), which is consistent with the results of the LVE parameters mentioned above.

#### 3.3.2. Microstructure Morphology Versus Rheological Performance

**Figure 10:**
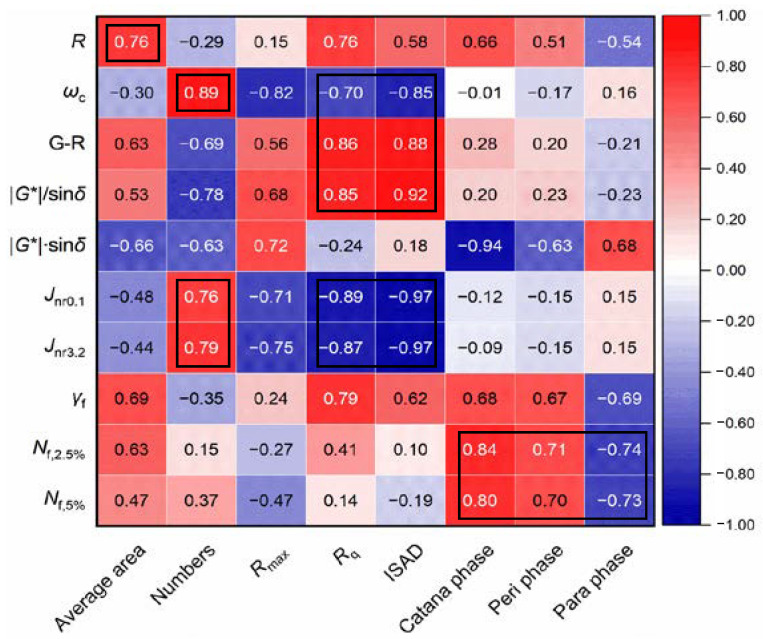
Correlations between morphology parameters and performance parameters. Note: For the Pearson correlation coefficient, the closer it is to 1 or −1, the stronger the linear correlation. A positive value means a positive correlation and a negative value means a negative correlation.

To comprehensively understand the influences of the bee structure and multiple phases on the rheological performance of asphalt binder, eight morphology parameters of three categories were selected and calculated. The test results are listed in Table 2. Further, the Pearson correlation analysis between morphology parameters and performance parameters were conducted and the Pearson correlation coefficients are summarized in Figure 10. It is observed that the average area of bee structures has a positive correlation with *R*, and the number of the bee structures also shows strong positive correlations with *ω*_c_, *J*_nr0.1_, and *J*_nr3.2_, indicating a potential relationship between the bee structure and the stiffness of asphalt binder. In addition, significant correlations (R > 0.8) between roughness-related parameters (*R*_q_ and ISAD) and LVE parameters (*ω*_c_, G-R and |G*|/sin *δ*) are observed. Seen from the trends of LVE parameters, the fracture resistance decreases and the rutting resistance increases with the increase in *R*_q_ and ISAD. The results indicate the LVE parameters are only affected by the surface structure of asphalt binder instead of material compositions or interactions between particles. This is expected since the LVE parameters are measured under a non-damaged state, where the contact between the plate and asphalt surface has a greater influence than the material itself. However, it is surprising to find that there are very significant correlations (R is around 0.9) in terms of the damage-based *J*_nr_ value vs. *R*_q_ and ISAD. The *J*_nr_ value increases with the increasing *R*_q_ and ISAD, indicating a weaker rutting potential, which is consistent with the change in |G*|/sin *δ*. Moreover, the content of the catana phase shows significant positive and negative correlations with fatigue life and |G*|·sin *δ*, respectively, which indicates the catana phase contributes significantly to the fatigue resistance nature of asphalt binder. In addition, the content of the peri phase and para phase also show a good correlation (R > 0.7) with fatigue life. The results prove that the fatigue resistance nature of asphalt binder contributes to the content of different compositions of asphalt binder rather than simple surface structure parameters since it is a damage-based index.

## 4. Conclusions and Recommendations

This paper presents comprehensive characterizations of linear viscoelastic (LVE) properties, damage resistance, and microstructure for seven unmodified asphalt binders from different sources but with the same penetration grade of 70. The specific findings of this study are summarized as follows:

(1) For the various LVE parameters, the crossover frequency of *ω*_c_ showed a significant correlation with |G*|/sin *δ* and G-R, with *R*^2^ higher than 0.90, and also exhibited a similar trend with |G*|/sin *δ*. However, there were no obvious correlations between the rheological index of *R* with other LVE parameters.

(2) For the LVE parameters and damage-based indexes, the correlation between the |G*|/sin *δ* vs. *J*_nr3.2_ and |G*|·sin *δ* vs. lower strain based-*N*_f_. was very promising (higher than 0.83 and 0.90 respectively), and the relationship between the rheological index of *R* and the LAS failure strain was also verified.

(3) Congregated and big bee structures formed on the surface of asphalt binder, resulting in stiffer, stronger, and more elastic properties. By comparison, for asphalt binders without the bee structure, a higher continuous light phase and lower dark quasi-spherical domains resulted in more viscosity and less rigidity and solidity in the asphalt, thus affecting the LVE properties of asphalt.

(4) The bee structure-related and roughness-related parameters were well correlated with LVE parameters, with the Pearson correlation coefficient ranging from 0.70 to 0.92; moreover the phase content was largely related to the material fatigue life (Pearson correlation coefficient higher than 0.70), indicating the initial material compositions have great impacts on the fatigue resistance nature.

In this paper, the possible relationship among various LVE parameters, damage-based indexes, and microstructure morphology parameters is proposed, which is beneficial for macro-index sections and macro–micro correlations. In the future, it is recommended to take more types of unmodified asphalt binders into account to further verify the relationship between rheological performance and microstructure morphology parameters.

## Figures and Tables

**Table 1:** Physical properties of all tested binders.

Technical Indexes	Binder Types						
	A	B	C	D	E	F	G
Penetration at 25 °C (0.1 mm)	66	67	68	67	71	67	81
Softening point (°C)	50.0	47.8	48.1	47.9	48.6	48.5	45.8
Ductility at 15 °C (cm)	>100	>100	>100	>100	>100	>100	>100

**Table 2:** Morphology parameters of tested asphalt binders.

Asphalt	Bee Structure		Roughness (nm)			Phase Content (%)		
	Average Area (μm^2^)	Numbers	*R*_max_	*R*_q_	ISAD (%)	Catana Phase	Peri Phase	Para Phase
A	0.411	35	37.97	3.47	0.057	3.60	43.00	53.40
C	0.353	49	27.87	2.11	0.019	3.28	37.62	59.10
D	0.368	45	31.81	2.86	0.033	4.14	50.67	45.18
E	0.254	38	37.57	1.82	0.023	2.41	41.64	55.95
F	0.367	47	28.86	2.30	0.017	4.32	50.54	45.14

Note: *R*_max_—difference between the maximum and minimum heights; *R*_q_—the high root mean square; ISAD—the image surface area difference.

## Data Availability

Not applicable.
